# Absolute Displacement-Based Formulation for Peak Inter-Story Drift Identification of Shear Structures Using Only One Accelerometer †

**DOI:** 10.3390/s21113629

**Published:** 2021-05-23

**Authors:** Kangqian Xu, Akira Mita

**Affiliations:** Department of System Design Engineering, Keio University, Yokohama 223-8522, Japan; xukangqian@keio.jp

**Keywords:** maximum inter-story drift, shear structure, modal coordinates, Kalman filter

## Abstract

Only one accelerometer is used in this paper for estimating the maximum inter-story drifts and time histories of the relative displacements of all stories of multi-degree-of-freedom (MDOF) shear structures under seismic excitation. The calculation based on the data of one sensor using a conventional method is unstable, and when modal coordinates are used, higher modes should be included, which is different from the estimation based on the responses recorded by many accelerometers. However, the parameters of the higher modes of structures are difficult to obtain from structures under small excitations. To overcome this difficulty, the recorded absolute acceleration is converted into the absolute displacement, and a state-space equation is formulated. Numerical simulations of a nine-story structure were conducted to check the applicability, robustness against environmental noise, and optimal installation location of the accelerometer of the proposed approach. In addition, the effects of the higher modes were analyzed in terms of the number of accelerometers and type of response. Finally, the proposed approach was validated in a simple experiment. The results indicate that it can accurately estimate the time histories of the relative displacements and maximum inter-story drifts of all floors when one accelerometer is used and just the first two modal parameters are incorporated in the model. Furthermore, the approach is robust against environmental noise.

## 1. Introduction

Structural health monitoring (SHM) technologies [1] have been extensively researched and used to assess structures. In particular, SHM technologies for identifying the structural parameters, state, and input [2,3,4] have received considerable attention, and numerous algorithms have been developed that use response data recorded by sensors.

The displacement of a structure under service loads can provide crucial safety and performance information. Since structural deformation and force are closely related, significant inter-story drift indicates large exogenous loads, that is, damage to structural or non-structural components that may have occurred during seismic motion. When nonlinear devices, such as isolated bearings or dampers, are employed, their characteristics behave differently, and hence, the modal parameters of the seismic isolation structures and supplementary-damped buildings vary under different structural displacements [5,6,7,8,9]. These mean that displacement identification is important in SHM.

Laser sensors can be used to detect displacements [10], but their placement requires a stationary reference, which is impractical in buildings. Vision-based displacement measurement [11] has been researched, but it is susceptible to visibility between the building and cameras and vibration of the cameras. In contrast, accelerometers are easy to deploy and usually have high accuracy. They can be used to record acceleration responses, from which the dynamic displacements can be mathematically calculated. In particular, double integration methods in the time domain or frequency domain [12] can convert the measured accelerations into displacement responses but are easily affected by low-frequency noise. To suppress the noise in the low-frequency band, finite impulse response filters (FIR filters) [13,14] have been studied by solving generalized minimization problems with Tikhonov regularization. In addition, Kalman filters [15,16] have been used to identify the structural displacement state from detected time histories of the acceleration responses for known or unknown inputs. When the superstructures experience loads, the recorded acceleration responses are relative to the ground, and hence, the relative displacements can be determined by the aforementioned schemes. However, these methods can only obtain the absolute displacement responses when the structures are excited by ground motion and the input signals are not gauged by sensors.

To deal with this problem, identifications using the absolute acceleration have been developed. Zhao et al. [17,18] proposed a hybrid method to identify structural parameters and seismic motion directly from measured absolute responses. However, this method requires the accelerations, velocities, and displacements of all floors, which limits its feasibility. Lei et al. [19,20] developed an extended Kalman estimator that requires the absolute acceleration responses of partial floors. Li et al. [21] and Huang et al. [22] modified this method to work in modal space in order to reduce the dimensions of state-space representation. These studies focused on the structural parameters or seismic wave, wherein the relative displacement can be obtained from the estimated absolute responses and input. On the other hand, the relative responses can be directly used to form the state vector based on the general equation of motion, which is formulated in a relative coordinate system, so that there is no direct feedthrough term in the state-space equation [23,24,25]. In addition, some researchers combined other responses data (e.g., displacement or strain) with acceleration responses to evaluate the structures [26,27,28,29]. Moreover, Oh et al. [30] used a convolutional neural network to predict the time histories of relative displacements from the recorded absolute accelerations, but relative error of the maxima (up to 16%) in the numerical simulation was unsatisfactory. Sun et al. [31] used kernel-based machine-learning methods to estimate the seismic response demands, such as peak inter-story drift. All of these algorithms require a lot of output data for training the model [30,31].

However, the installation of many sensors is laborious and time consuming, and complicated sensing networks are expensive to maintain. For these reasons, simple SHM systems are favored over complicated ones. Thus, the identification algorithms that use limited output measurements [19,20,21,22,23,24,25] have been developed to simplify the sensing systems of Zhao et al.’s method [17,18] (it requires sensors to be deployed on all floors). Nevertheless, these methods [19,20,21,22,23,24,25] still entail more than one accelerometer to provide sufficient output data, and in particular, the methods in the literature [22,23,26,27,28,29] require simultaneous measurements from accelerometers and displacement or strain sensors for data fusion. To simplify SHM systems further, this study develops an identification algorithm for determining the relative displacement from measurement made by one accelerometer.

The method presented in this study focuses on the relative displacement responses and peak inter-story drifts. It uses modal coordinates instead of physical coordinates [19,20] whose use is inadvisable when other structural parameters are unavailable and there are few output data. Moreover, the practicability of the estimation should be considered when the excessively limited output measurements are available. The responses can be precisely evaluated in modal coordinates by using a model including the first few modes [21,22], but their accuracy will deteriorate as the measurements decrease. In particular, the authors have tried to identify the relative displacement directly from the measured absolute acceleration by using only one accelerometer [32]. When high-rise buildings are evaluated, more modes are required in the model when the estimation is based on single observation data. However, the modal parameters of most reinforced concrete and steel shear structures are difficult to identify, because the frequencies of the higher modes are large and hence usually out of the effective range of the power spectral density (PSD) of common natural loads such as wind or weak seismic motion. To evaluate the relative displacement of the structures under seismic motion from measurement made by one accelerometer by using only a few modal parameters, the absolute acceleration is converted into the absolute displacement response, and subsequently, the maximum inter-story drift is estimated in modal coordinates.

This paper is organized as follows: Section 2 formulates the proposed algorithm and briefly introduces the Kalman filter with unknown input. Section 3 describes numerical simulations to investigate the feasibility, robustness against environmental noise, and optimal installation location of the accelerometer of the proposed approach. It also makes a comparison with previous methods and analyzes the contribution of high modes. Section 4 validates the proposed method in a shaking-table experiment. Section 5 concludes this study and indicates the potential of this research.

## 2. Formulation of Proposed Method

The literature [21] indicated that the estimation is vulnerable to the errors in identified natural frequencies. Since we aim at using just the first few modes to assess the multi-degree-of freedom (MDOF) structure, we will assume that the modal parameters of these lower modes of the structure, including its natural frequencies, damping ratios, and mode shapes, can be accurately identified under ambient excitation. The proposed approach estimates the relative displacement and inter-story drift of the structure by using one accelerometer and a few modes in modal coordinates when the structure is excited by a seismic motion.

**Figure 1:**
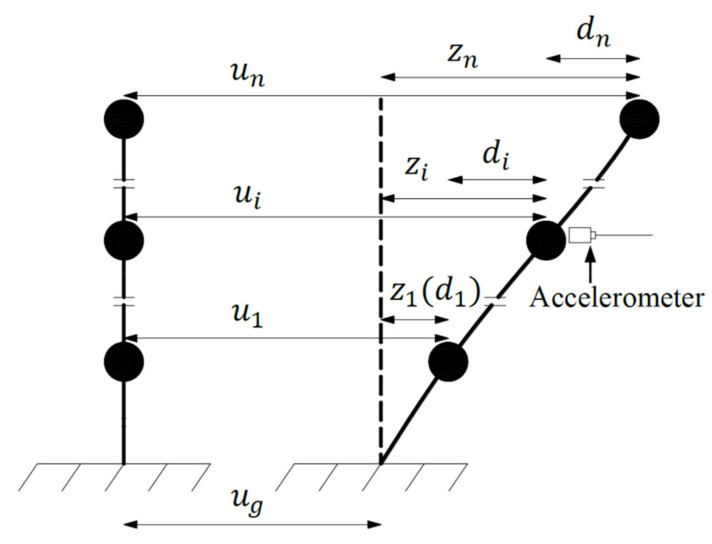
MDOF structure subjected to seismic excitation.

As shown in Figure 1, the equation of motion of a structure that is subjected to a seismic excitation can be described as:

(1) $$M\ddot{z}(t)+C\dot{z}(t)+Kz(t)=-Mr{\ddot{u}}_{g}(t)$$

where z(t) represents the displacement relative to the ground, (⋅) means the time derivative, ${\ddot{u}}_{g}(t)$ is the seismic acceleration, r denotes an n×1 unit vector $\left(r={\left[\begin{array}{ccc} 1 & \cdots & 1 \end{array}\right]}^{T}\right)$, and M, C, and K are respectively the structural mass, damping, and stiffness matrices.

A simple sensing system is employed, that is, merely one accelerometer is placed on a floor, which greatly reduces the maintenance cost. Accordingly, when an earthquake occurs, only the time history of the absolute acceleration of the floor on which the accelerometer is placed can be obtained. The problem is that an identification made from acceleration data of one sensor may be inaccurate when higher modes are truncated. On the other hand, higher modes contribute comparatively less to the displacement response than to the acceleration response, so a model composed of a few modes is feasible when a single output of displacement responses is available. In this case, the time history of the absolute displacement $u_{i}(t)$ is used to estimate the maximum relative displacement $z{\left(t\right)}_{\max}$ and subsequently the inter-story drift $d{\left(t\right)}_{\max}$. The absolute displacement $u_{i}(t)$ can be calculated from the recorded absolute acceleration ${\ddot{u}}_{i}(t)$ [12,13].

The relative displacement of the ith DOF of the MDOF structure can be expressed in modal coordinates as

(2) $$z_{i}(t)=\sum_{j=1}^{n}\phi_{ij}\eta_{j}(t)\approx \sum_{j=1}^{m}\phi_{ij}\eta_{j}(t)$$

$$\Phi =\left[\begin{array}{ccccc} \phi_{1} & \cdots & \phi_{j} & \cdots & \phi_{n} \end{array}\right]=\left[\begin{array}{ccccc} \phi_{11} & \cdots & \phi_{1j} & \cdots & \phi_{1n}\\ \vdots & \ddots & \vdots & \ddots & \vdots \\ \phi_{i1} & \cdots & \phi_{ij} & \cdots & \phi_{in}\\ \vdots & \ddots & \vdots & \ddots & \vdots \\ \phi_{n1} & \cdots & \phi_{nj} & \cdots & \phi_{nn} \end{array}\right]$$

where Φ denotes the mode shape matrix of the structure, η(t) is the modal coordinate, n is the total number of DOFs of the structure, m (m≪n) represents the number of modes to be considered, the subscript i means the DOF whose response is focused on, and the subscript j indicates the order of the mode.

Hence, the absolute displacement of the ith DOF can be expressed in modal coordinates as

(3) $$\begin{array}{cc} u_{i}(t) & =z_{i}(t)+u_{g}(t)\\ & =\sum_{j=1}^{n}\phi_{ij}\eta_{j}(t)+u_{g}(t)\\ & \approx \sum_{j=1}^{m}\phi_{ij}\eta_{j}(t)+u_{g}(t) \end{array}$$

The modal displacement response $\eta_{j}(t)$, modal velocity response ${\dot{\eta }}_{j}(t)$, ground displacement $u_{g}(t)$, and ground velocity ${\dot{u}}_{g}(t)$ is used to constitute the state vector:

$$x_{k}={\left[\begin{array}{cc} x_{k}^{\eta } & x_{k}^{g} \end{array}\right]}^{T}={\left[\begin{array}{cccccccc} \eta_{1}(k) & {\dot{\eta }}_{1}(k) & \cdots & \cdots & \eta_{m}(k) & {\dot{\eta }}_{m}(k) & u_{g}(k) & {\dot{u}}_{g}(k) \end{array}\right]}^{T}$$

As for the modal responses, Rayleigh damping, i.e., proportional viscous damping, is adopted, and the mode shape matrix is normalized using

(4) $$M^{*}=\Phi^{T}M\Phi =I_{}$$

where the superscript * represents the corresponding matrix in modal coordinates, and I is an identity matrix of appropriate dimension.

The equation of motion in modal coordinates can be simplified as

(5) $${\ddot{\eta }}_{j}(t)+2\xi_{j}\omega_{j}{\dot{\eta }}_{j}(t)+\omega_{j}^{2}\eta_{j}(t)=-\phi_{j}^{T}Mr{\ddot{u}}_{g}(t),\;j=1,2\cdots m$$

Thus, the state-space equation of the modal responses in continuous time can be formulated as

(6) $${\dot{x}}_{k}^{\eta }=A_{c}^{\eta }x_{k}^{\eta }+B_{c}^{\eta }p_{k}+w_{k}^{\eta x}$$

$$A_{c}^{\eta }=\left[\begin{array}{cccccc} 0 & 1 & & & & 0\\ -\omega_{1}^{2} & -2\xi_{1}\omega_{1} & & & & \\ & & \cdots & \cdots & & \\ & & \cdots & \cdots & & \\ & & & & 0 & 1\\ 0 & & & & -\omega_{m}^{2} & -2\xi_{m}\omega_{m} \end{array}\right]$$

$$B_{c}={\left[\begin{array}{cccccc} 0 & -1 & \cdots & \cdots & 0 & -1 \end{array}\right]}^{T}$$

The discrete state-space matrices can be calculated on the basis of the above matrices in continuous time:

(7) $$\begin{array}{c} A^{\eta }=\exp(A_{c}^{\eta }\Delta t)\\ B^{\eta }=(A_{c}^{\eta }-I){\left(A_{c}^{\eta }\right)}^{-1}B_{c}^{\eta } \end{array}$$

The ground acceleration, velocity, and displacement can be calculated using numerical integration [33]. Therefore, the state-space equation of the ground motion in discrete time can be expressed as

(8) $$x_{k}^{g}=A^{g}x_{k}^{g}+B^{g}p_{k}+w_{k}^{gx}$$

$$A^{g}=\left[\begin{array}{cc} 1 & \Delta t\\ 0 & 1 \end{array}\right]\;B^{g}={\left[\begin{array}{cc} \Delta t^{2}/2 & \Delta t \end{array}\right]}^{T}$$

The total state-space equation in discrete time is constituted from the state-space representations of the modal responses and the ground motion:

(9) $$x_{k+1}=Ax_{k}+Bp_{k}+w_{k}^{x}$$

$$A=\left[\begin{array}{cc} A^{\eta } & 0\\ 0 & A^{g} \end{array}\right]\;B={\left[\begin{array}{cc} {\left(B^{\eta }\right)}^{T} & {\left(B^{g}\right)}^{T} \end{array}\right]}^{T}\;w_{k}^{x}={\left[\begin{array}{cc} {\left(w_{k}^{\eta x}\right)}^{T} & {\left(w_{k}^{gx}\right)}^{T} \end{array}\right]}^{T}$$

The measurement equation can be derived from Equation (3):

(10) $$y_{k}=Hx_{k}+v_{k}$$

$$H={\left[\begin{array}{cccccccc} \phi_{i1} & 0 & \cdots & \cdots & \phi_{im} & 0 & 1 & 0 \end{array}\right]}^{T}$$

where $p_{k}$ is an unknown ground input, and Δt is the sampling time. The process noise $w_{k}^{vx}$, $w_{k}^{gx}$, and measurement noise $v_{k}$ are assumed to be white, zero-mean, and uncorrelated with known covariance matrices $Q^{vx}$, $Q^{gx}$, and R, respectively.

The state and input of the state-space equation without the direct feedthrough term can be estimated by using variants of Kalman filters [16,34,35,36]. These variants have their advantages and disadvantages, which means that their applicability may vary from one situation to another. It is reported that dual filters can reduce the low-frequency drift of the estimated input and state, which may happen when the input to the state vector augmented [36,37]. As the proposed method focuses on the peak relative displacement and hence requires a reliable state estimation, a dual filter was chosen in which one of the filters is used to estimate the input based on a Gaussian random walk model. The resulting algorithm is listed in Table 1, and the L-curve [37] is used to tune the covariance of input.

The relative displacements of all floors can be directly determined from the estimate:

(11) $$z_{k}=Lx_{k}$$

$$L=\left[\begin{array}{cccccc} \phi_{11} & 0 & \cdots & \cdots & \phi_{1m} & 0\\ \phi_{21} & 0 & \cdots & \cdots & \phi_{2m} & 0\\ \vdots & \vdots & \ddots & \ddots & \vdots & \vdots \\ \phi_{n1} & 0 & \cdots & \cdots & \phi_{nm} & 0 \end{array}\right]$$

**Figure 2:**
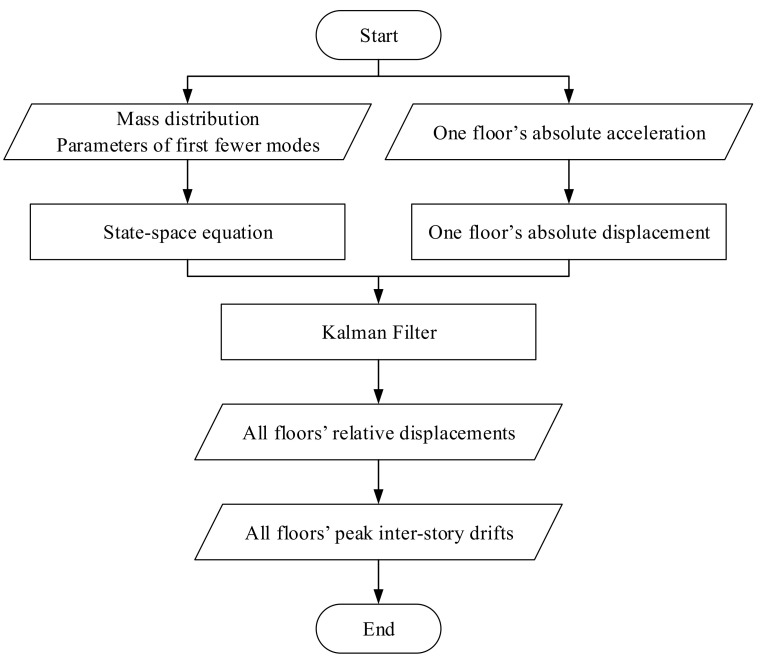
Procedure of the proposed algorithm.

If the filter can accurately determine the structural state, consequently, the high precision will be observed in estimated relative displacement. In other words, this method will be able to accurately estimate first floor’s inter-story drift, which is equal to the relative displacement of the floor. As well, the maximum horizontal deformation of the building can be determined from the relative displacement of the top floor. The inter-story drifts of the other stories are computed from the estimated displacement responses of the adjacent floors. This process amplifies the errors in the drifts of these stories. A flow chart of the estimation process is shown in Figure 2.

## 3. Numerical Simulation

Numerical simulations were conducted to verify the proposed approach. Section 3.1 investigates the applicability of our method when only the parameters of the first two modes are available. Section 3.2 compares the proposed method with the previous algorithms and analyzes the effect of high modes on the estimation result in terms of the number of measurements and type of responses. Section 3.3 discusses the robustness of the method against noise. Section 3.4 deals with the optimal installation location of the single accelerometer.

### 3.1. Results of Proposed Method

**Figure 3:**
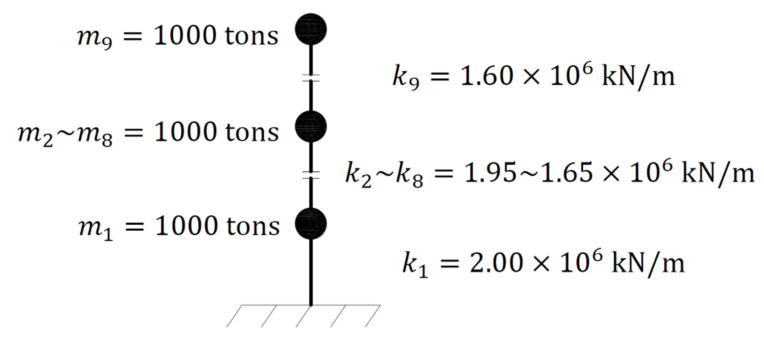
Simulation model of nine-story structure.

Numerical simulations of a nine-story shear structure were performed. As shown in Figure 3, the lumped masses of all floors were each 1000 tons. The lateral inter-story stiffness of each floor was linearly varied from $2.00\times 10^{6}\;\mathrm{kN}/m$ to $1.60\times 10^{6}\;\mathrm{kN}/m$ as the height of floor increased. The first two natural frequencies were 1.13 and 3.31 Hz. The damping ratios of the first and second modes were 2% and 3%, respectively, and the damping ratios of the other modes obeyed Rayleigh damping.

The El Centro earthquake wave (NS component), a typical seismic acceleration, was used to excite the structure, and the time histories of the structural responses were computed by the Newmark beta method [38]. The accelerometer was installed on the first floor, and only the parameters of the first two modes were known. The measurement noise R and the process noise of the state $Q^{vx}$ and $Q^{gx}$ were respectively set to $10^{-2}I$, $10^{-4}I$, and $10^{-4}I$ (I is an identity matrix of appropriate dimension). The covariance of the input $Q^{p}=10^{10}I$ was chosen by using the L-curve method. The absolute displacement was calculated from the absolute acceleration time history in two ways: (i) a four-order high-pass Butterworth filter was applied to the recorded response to remove low-frequency noise, and subsequently, double integration was implemented in the frequency domain; (ii) an FIR filter [13] based on a generalized minimization problem with Tikhonov regularization was utilized.

**Figure 4:**
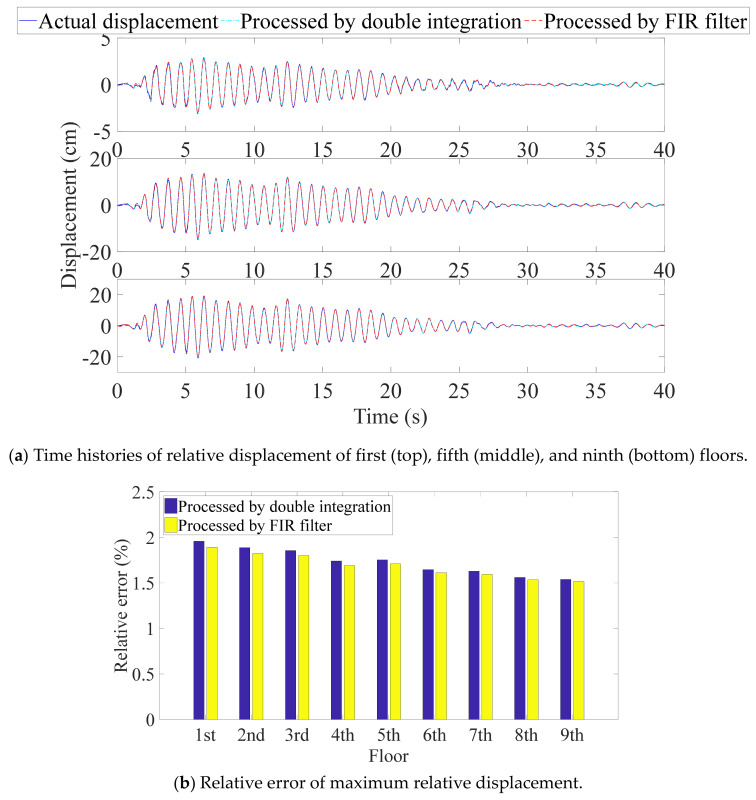
Estimated relative displacement of nine-DOF structure.

**Figure 5:**
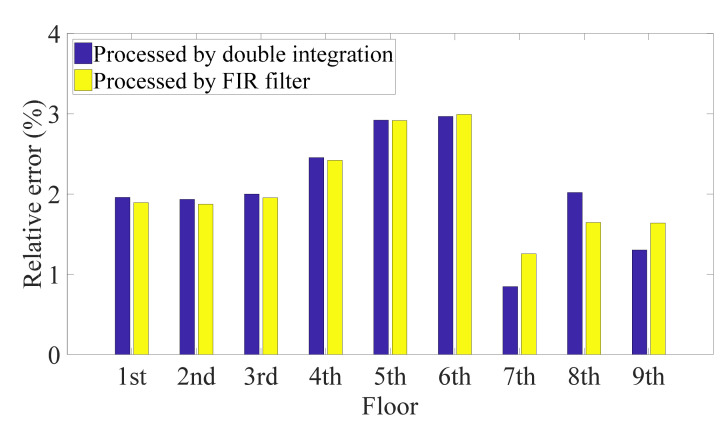
Relative error of maximum inter-story drift of nine-DOF structure.

The proposed algorithm estimated the relative displacements and inter-story drifts from the single absolute displacement response. Taking the first, fifth, and ninth floors as examples, Figure 4a displays superior recoveries of the whole time history responses by the model including the first two modes. Moreover, as shown in Figure 4b, the insignificant errors of the maximum relative displacements of all floors indicate a good estimation of structural deformation, i.e., reliable evidence for making a structural assessment. In addition, the variance of the results, 0.022 (for double integration), reveals consistent estimation performance for all floors, even though the sensor was deployed on the first floor. Figure 5 indicates that although the peak inter-story drifts can be obtained, the errors fluctuate more (i.e., with a variance of 0.470 for double integration) compared with the relative displacements, because errors accumulate when the drifts are determined from the displacement responses of the adjacent floors. Furthermore, the figure shows that the proposed method gives similar estimates of the relative displacement and inter-story drift when different algorithms are used to compute the absolute displacement from the acceleration. The case with permanent deformation is not considered, as the permanent deformation is very easy to identify by simple visual inspection. If permanent deformation is excluded, the double integration with proper filters usually works well. In addition, real-time estimation of the maximum relative displacement is not necessary. Therefore, the use of double integration in combination with a high-pass filter is preferred because the FIR filter requires more parameters.

### 3.2. Comparison with Previous Research and Analysis of the Contribution of High Modes

**Figure 6:**
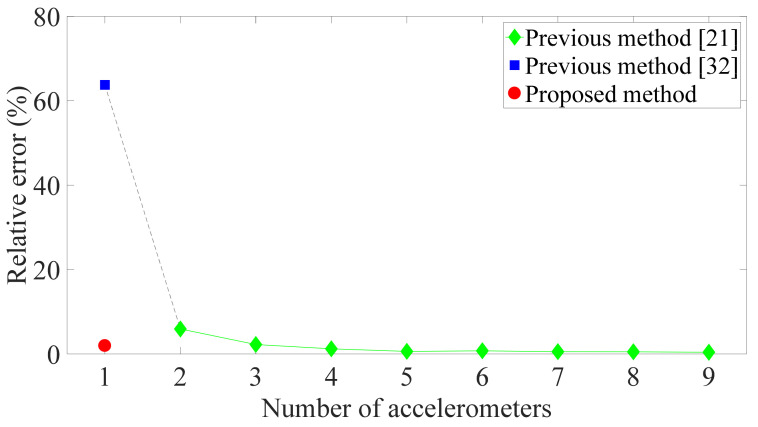
Relative error of first floor’s peak inter-story drift of nine-DOF structure for the method in [21] (which uses two or more accelerometers), method in [32] (one accelerometer), and proposed method (one accelerometer) when only two modes are included in the model.

In previous research, identification algorithms are developed based on the measurements of many sensors, and the structural responses, parameters, and input attract lots of interest. Different focuses of identification interest correspond to different approaches. For instance, the methods in [19,20] put the emphasis on the evaluation of structural parameters and consequently formulate the model in physical coordinates. Since the identification of relative displacement and inter-story drift are the focus of this research, modal coordinates are employed in the proposed algorithm to circumvent the estimation of unknown structural stiffness. Therefore, the comparison is made for the algorithms using modal coordinates. In this comparison, only the first two modes were included in the proposed methods and the compared methods. The compared method in [21] was used to calculate the relative displacements from the absolute acceleration responses for different numbers of accelerometers, while the method in [32] was used when merely one accelerometer was deployed, because the method reported in [21] will have trouble calculating the absolute modal acceleration response in this case. The results for the first floor are shown in Figure 6. It can be seen that the maximum inter-story drift is accurately estimated by the method in [20], when there are measurements from three or more accelerometers, while its estimation accuracy deteriorates slightly when there are only two accelerometers. Moreover, the relative error of the method in [32] is huge when the displacement is calculated only from data of one sensor. In contrast, the proposed algorithm precisely estimates the inter-story drift by using data from one accelerometer when only two modes are included in the model.

**Figure 7:**
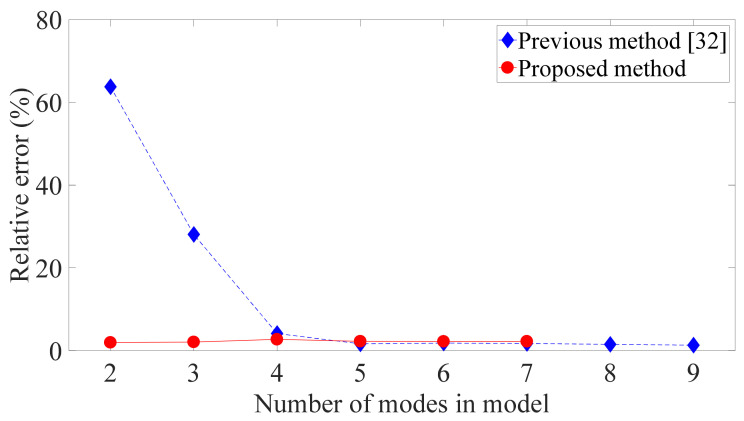
Relative error of first floor’s peak inter-story drift of nine-DOF structure for different numbers of modes in model when only one accelerometer is used.

To investigate the effect of including higher modes in the model when one accelerometer is used, the error in the relative displacement was determined when different numbers of modes were included in the method in [32] and the proposed method. The method in [32] directly treats the absolute acceleration as observation in the Kalman filter, and as shown in Figure 7, it accurately evaluates the inter-story drift of the first floor when the first four modes are included in the model. However, its error dramatically increases when fewer modes are incorporated. Thus, when recorded acceleration responses are directly used in the filtering process, the estimation made from one absolute acceleration is susceptible to truncation error of higher modes, that is, more modes (i.e., no less than four modes) should be taken into account in the case of using one accelerometer than in the case that the estimation is made from the measurements of many sensors (i.e., two modes is enough). On the other hand, the proposed method can precisely determine the inter-story drift using the first two modes, and its accuracy remains basically the same when the third to seventh modes are added to the model. It should also be noted that including too many high modes in the model (i.e., including more than seven modes) would induce numerical instabilities due to their tiny amplitudes, so the data of the proposed method for eight and nine modes in Figure 7 is vacant. In actuality, the higher modes of most reinforced concrete and steel shear structure are difficult to excite under wind or small seismic loads, because their natural frequencies are out of the effective frequency band of these natural loads, and hence, the corresponding modal parameters cannot be identified. Therefore, a model using modal coordinates should not incorporate too many modes from a practical standpoint, which can be satisfied by using the proposed method when only the output response of one floor is detected.

The effect of varying the number of modes in the model was quantitatively analyzed by using the proper orthogonal decomposition (POD) technique [21,22,39]. In this technique, the eigenvalues are calculated from the covariance matrix of the structural acceleration or displacement responses, and they give the energy contribution of each mode to the corresponding responses. The energy contributions are listed in Table 2. The mode selection criterion in the literature [21,22] for the acceleration responses is 95% or more for the sum of the energy contributions. As shown in Table 2, the energy sum of first two modes to the absolute acceleration is 98.92%, which is enough to meet this criterion. This is consistent with the results of the method in [21] in Figure 6. However, this criterion is just suitable for the case of numerous measurements. In the circumstance where the estimation is made from the data of one accelerometer, at least four modes are required in the model, and the energy sum of these modes is more than 99%. Thus, a relatively bigger threshold (i.e., more than 99%) is necessary when merely the response of one floor is available for the displacement identification. In contrast, the table shows that from the viewpoint of the absolute displacement, the first two modes contribute virtually all of the energy. Therefore, the criterion for the single output measurements can be easily satisfied by using first two modes when the absolute displacement response is used in the Kalman filter.

### 3.3. Discussion of Environmental Noise

**Figure 8:**
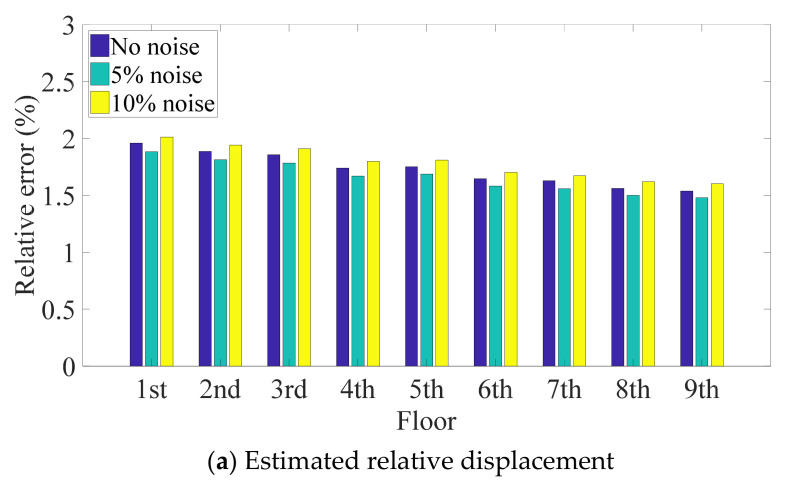

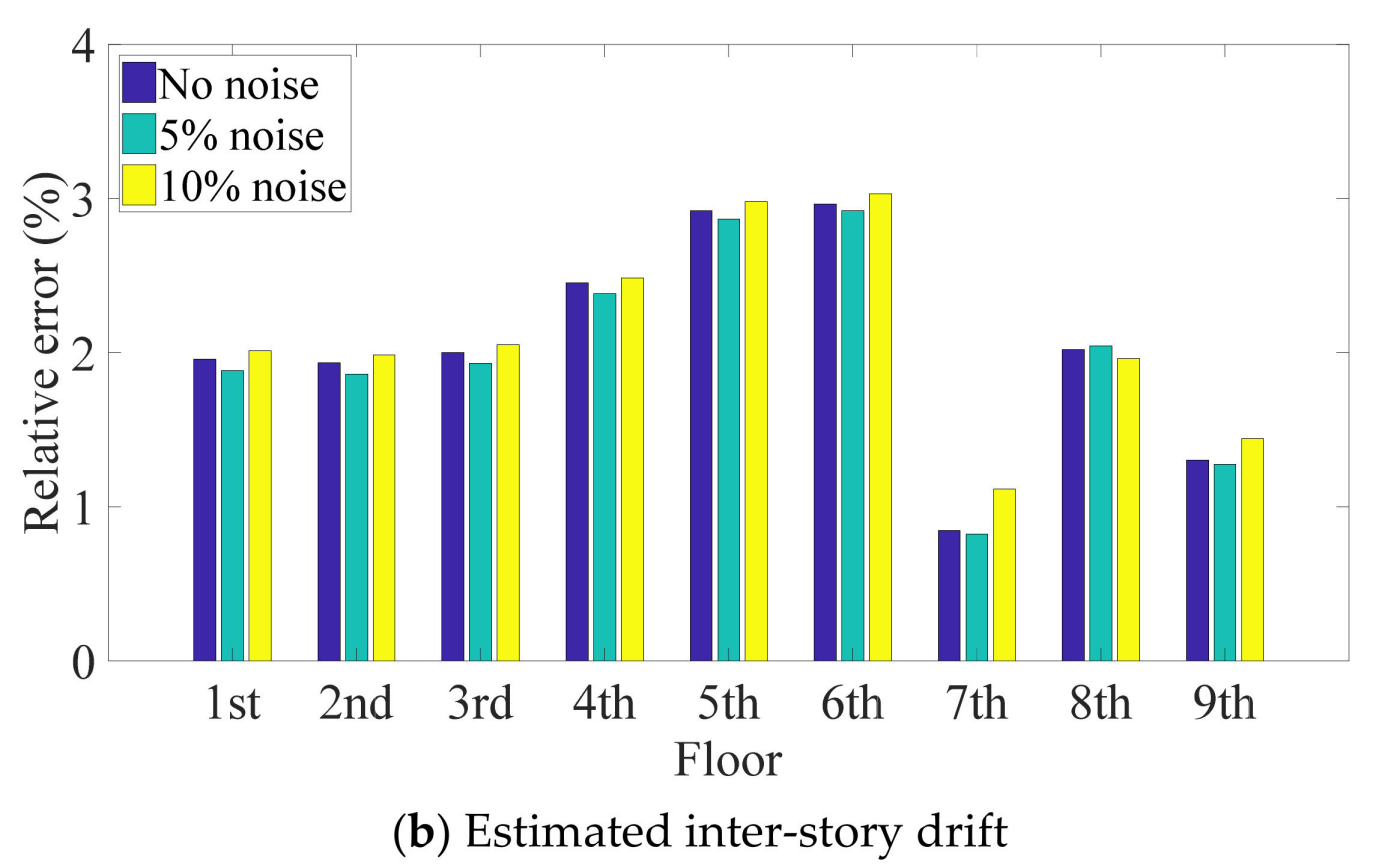
Relative maximum error of nine-DOF structure for different noise levels.

In practice, structural responses detected by sensors contain environmental noise. Thus, noise in the recorded acceleration data affects the acquisition of the absolute displacement and may cause the performance of the Kalman filter to decline. To investigate the robustness of the proposed method to environmental noise, a simulation was conducted in which Gaussian white noise was added to the absolute acceleration responses of a nine-story structure under excitation from the El Centro earthquake at 5% and 10% levels of the signal RMS. First, the noise was generated at random, and then the proposed technology was used to compute the relative displacement from the polluted response. As a result of the randomness of noise generation, this procedure was repeatedly implemented under the same noise level in order to ensure the reliability of estimation, and the average relative errors are plotted in Figure 8. The two plots show the same trend; that is, environmental noise levels of 5% and 10% have little effect on the estimated relative displacement and inter-story drift. This is mainly because the low-frequency noise was removed by the high-pass filter, and the magnitude of the high-frequency noise was reduced in the process of double integration. In addition, the errors of the estimated relative displacements of each floor are more uniform than those of the inter-story drift, which is consistent with the conclusion in Section 4.1. In addition, when the structure is subject to a seismic load and other environmental excitations simultaneously, in general, the amplitude of the response induced by other ambient excitations is less than that caused by the earthquake, and hence, ignoring these secondary responses will have a little effect on the results. If the structure suffers from a small earthquake so that other ambient vibrations cannot be neglected, more accelerometers are required to obtain accurate results.

### 3.4. Discussion of Installation Location of Accelerometer

**Figure 9:**
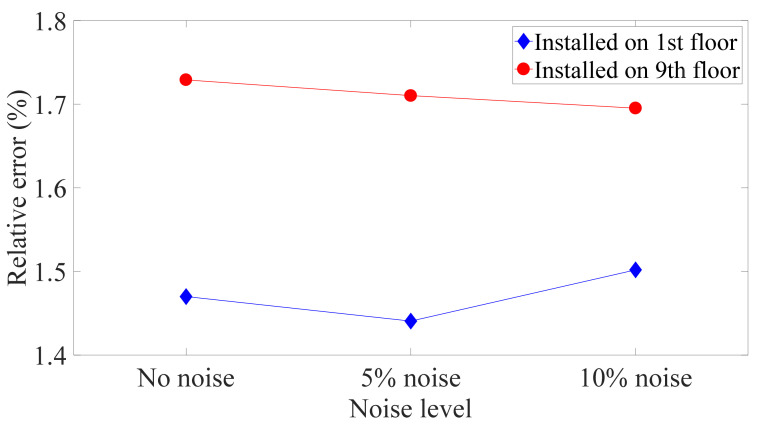
Relative error of estimated drift of ninth floor of nine-DOF structure for different installation locations and noise levels.

Although the simulations described above demonstrated that the relative displacements and inter-story drifts of all floors can be identified by deploying one accelerometer on the first floor, a question arises in the case that a middle or top story should be paid attention in a special circumstance: Will the estimation be improved by installing the sensor on the target story? To study the impact of the installation location of the accelerometer, the case of placing an accelerometer on the top floor was examined. As shown in Figure 9, the evaluation for the top floor is not improved by installing the accelerometer there. Instead, the estimation accuracy slightly deteriorates, which is mainly because large differences in the contributions of the different modes to the responses of high floors cause the values of the state vector to differ greatly, and consequently, the solution of the Kalman filter is prone to a large error. On the other hand, the ground floor of a shear structure usually suffers from the largest horizontal load under seismic motion, and hence, significant inter-story drift appears on the first story. Therefore, by placing the accelerometer on the first floor, the inter-story drift of the floor can be accurately obtained, which can meet most of the requirements in SHM.

## 4. Experimental Verification

**Figure 10:**
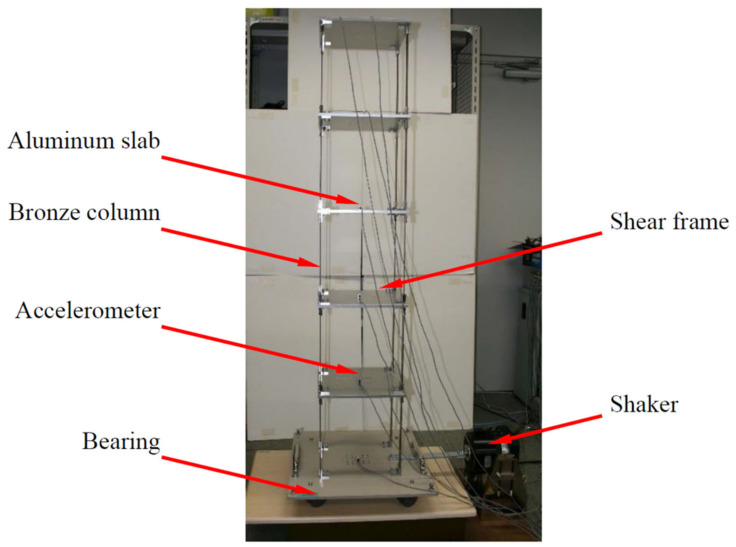
Setup of experiment on five-story shear frame structure.

To check the practicability of the proposed algorithm, a simple shaking-table experiment imitating a five-story shear frame structure was performed at the Mita Laboratory of Keio University. As exhibited in Figure 10, the floor of the structure was composed of an aluminum slab, and four bronze columns rigidly connected to the slab. Bearings were fitted under the base so that the shear structure could be excited by the shaker. The lumped masses of the first to fourth floors were each 4.360 kg, and that of the fifth floor was 3.544 kg. The lateral inter-story stiffness calculated from the size and Young’s modulus of the bronze column was $1.356\times 10^{4}$ N/m for each story.

The frame structure was excited by a sinusoidal wave whose frequency was swept from 1.0 to 20.0 Hz. Six accelerometers were deployed on the base and each floor to record the time histories of input and structural responses at a sample frequency of 100 Hz. To reduce the effect of high-frequency noise, a four-order low-pass Butterworth filter with a cut-off frequency of 30 Hz was applied to the measured data. Then, the combined deterministic-stochastic subspace identification method [40] was used to identify the eigenfrequencies and eigenvectors from the input and five responses. The identified natural frequencies were 2.50, 7.84, 12.47, 16.10, and 18.45 Hz for the first to fifth modes. In addition, the relative displacements of the floors were solved by integrating the acceleration responses after applying a high-pass filter.

### 4.1. Results and Discussion

**Figure 11:**
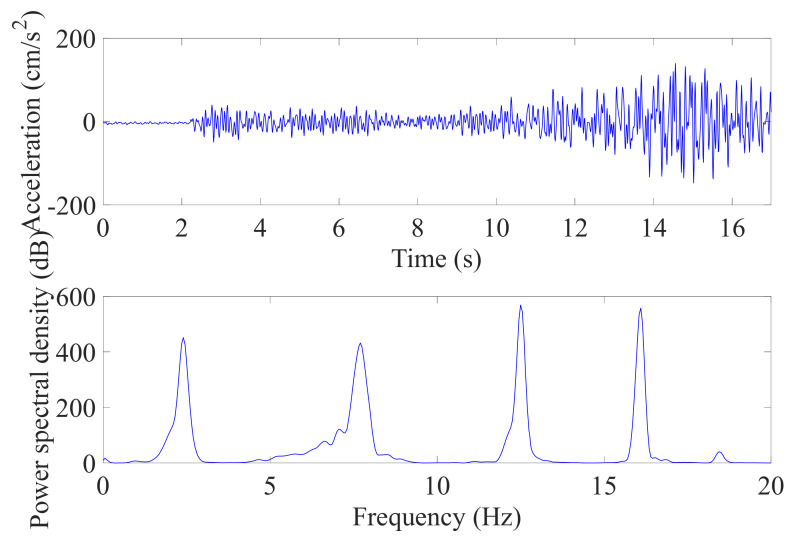
Absolute acceleration response and power spectral density of first floor.

**Figure 12:**
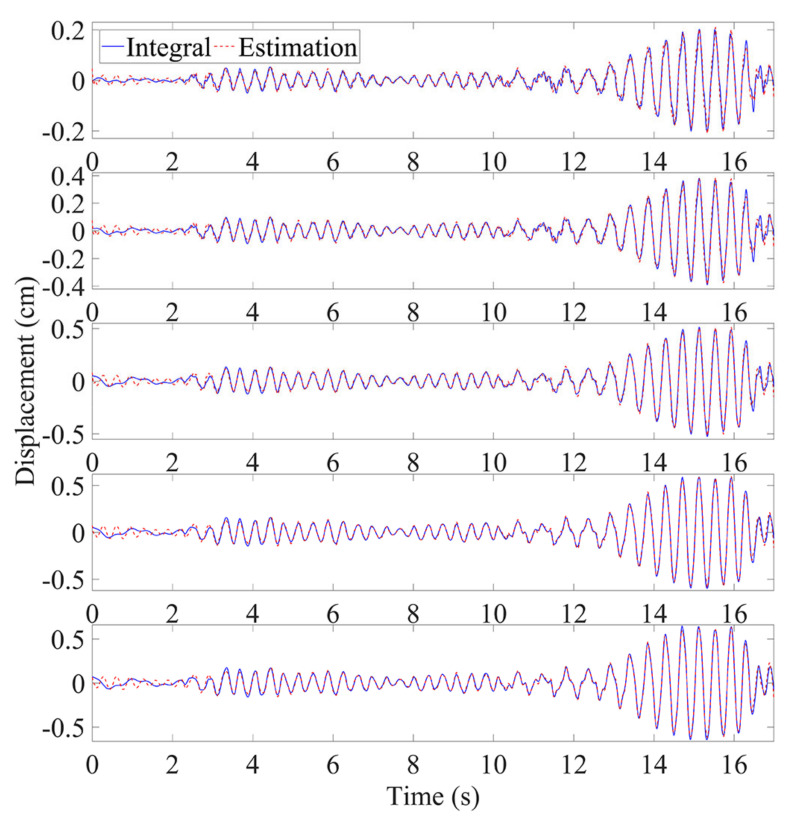
Time histories of relative displacement of five-story frame (first to fifth floor from top to bottom).

**Figure 13:**
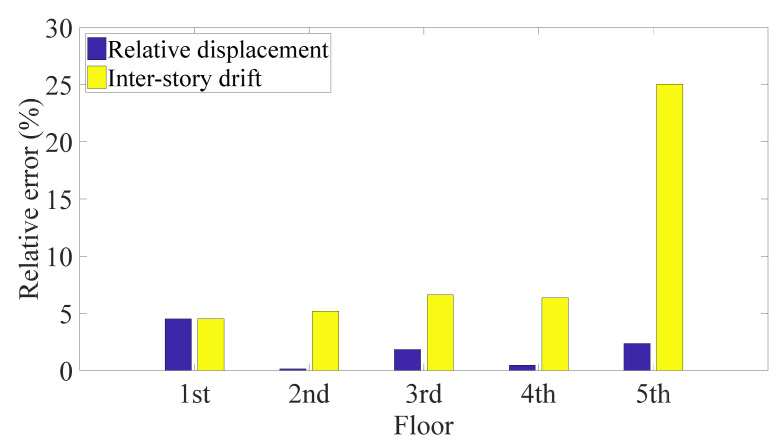
Relative error of peak relative displacement and inter-story drift of five-story frame.

Here, it was supposed that only the parameters of the first two modes were available and the first floor’s absolute response was detected. The measured response of the first floor and corresponding PSD are plotted in Figure 11, and the energy contributed by each mode is listed in Table 3. It can be observed that higher modes were excited by the artificial signal and they contributed relatively more energy to the acceleration responses. The recorded acceleration of the first floor was converted into an absolute displacement response by using a high-pass filter and double integration, and then, the relative displacements of each floor was evaluated from the absolute displacement. As shown in Figure 12, the time histories of the relative displacements were precisely estimated. Moreover, as shown in Figure 13, the maximum displacements had acceptable errors. The results confirm that the estimation using the absolute displacement is a good way to reduce the size of the model even though in this experiment first two modes just contributed the energy of 76.07% from the viewpoint of the absolute acceleration. The actual peak inter-story drifts of the first to fifth floors were 0.20, 0.18, 0.12, 0.07, and 0.04 cm, respectively. As illustrated by the relative errors of the estimated inter-story drifts in Figure 13, the inter-story drifts of the first four floors were accurately identified, while the estimated inter-story drift of the top floor, which had a minor deformation (i.e., 0.04 cm), showed a large error. In structural assessment, since the maximum deformation usually receives the most attention, the relatively larger deviations in the higher floors are not crucial. In summary, the proposed method precisely identified the time histories of the relative displacements of all floors and gave a good estimate of the inter-story drifts of the floors with significant deformation when only first few modes were incorporated in model. In addition, the double integration method can still figure out the results with acceptable accuracy in this experiment, which is consistent with the conclusion in Section 3.1. Nevertheless, if the application environment is harsher, the FIR filter will be a more appropriate selection, because it will perform better to obtain the absolute displacement response than double integration.

## 5. Conclusions

The proposed method estimates the time history of the relative displacement and maximum inter-story drift of MDOF shear structures under seismic excitation by using one accelerometer and the first two modes. The state-space equation is based on the absolute displacement derived from the recorded acceleration response. Simulations and experiments showed that proposed method obtains accurate estimates of the relative displacement and maximum inter-story drift from data of one sensor by using the first two modes and that its estimation performance only slightly deteriorates in noisy environments. In addition, its accuracy is competitive with those of previous methods for assessing the states of structures under the restriction of using only one accelerometer and the first few modes.

## Figures and Tables

**Table 1:** Two-stage filter for the input and state estimation.

1. Initialization $x_{0|0}$, $P_{0|0}^{x}$, $p_{-1}$, $P_{-1|-1}^{p}$, $Q^{x}$($Q^{vx}$, $Q^{gx}$), $Q^{p}$, R 2. Time update of input $p_{k|k-1}=p_{k-1|k-1}$ $P_{k|k-1}^{p}=P_{k-1|k-1}^{p}+Q^{p}$ 3. Measurement update of input $G_{k}^{p}=P_{k|k-1}^{p}{\left(HB\right)}^{T}{\left[(HB)P_{k|k-1}^{p}{\left(HB\right)}^{T}+R\right]}^{-1}$ $p_{k|k}=p_{k|k-1}+G_{k}^{p}(y_{k+1}-HAx_{k|k}-HBp_{k|k-1})$ $P_{k|k}^{p}=(I-G_{k}^{p}B)P_{k|k-1}^{p}$ 4. Time update of state $x_{k+1|k}=Ax_{k|k}+Bp_{k|k}$ $P_{k+1|k}^{x}=AP_{k|k}^{x}A^{T}+Q^{x}$ 5. Measurement update of state $G_{k+1}^{x}=P_{k+1|k}^{x}H^{T}{\left(HP_{k+1|k}^{x}H^{T}+R\right)}^{-1}$ $x_{k+1|k+1}=x_{k+1|k}+G_{k+1}^{x}(y_{k+1}-Hx_{k+1|k})$ $P_{k+1|k+1}^{x}=(I-G_{k+1}^{x}H)P_{k+1|k}^{x}$

**Table 2:** Energy contribution of different modes of nine-DOF structure.

Order	Absolute Acceleration		Absolute Displacement	
	Energy Contribution (%)	Summation (%)	Energy Contribution (%)	Summation (%)
1	94.81	94.81	99.94	99.94
2	4.11	98.92	0.06	100.00
3	0.79	99.71	0.00	100.00
4	0.22	99.93	0.00	100.00
5	0.06	99.99	0.00	100.00
6	0.01	100.00	0.00	100.00

**Table 3:** Energy contribution of different modes of five-story frame.

Order	Absolute Acceleration		Absolute Displacement	
	Energy Contribution (%)	Summation (%)	Energy Contribution (%)	Summation (%)
1	57.94	57.94	99.92	99.92
2	18.57	76.07	0.07	99.99
3	12.18	88.24	0.01	100.00
4	10.21	98.46	0.00	100.00
5	1.54	100.00	0.00	100.00
